# Should prenatal chromosomal microarray analysis be offered for isolated ventricular septal defect? A single-center retrospective study from China

**DOI:** 10.3389/fcvm.2022.988438

**Published:** 2022-09-07

**Authors:** Ken Cheng, Hang Zhou, Fang Fu, Tingying Lei, Fucheng Li, Ruibin Huang, You Wang, Xin Yang, Ru Li, Dongzhi Li, Can Liao

**Affiliations:** ^1^School of Medicine, South China University of Technology, Guangzhou, China; ^2^Prenatal Diagnostic Center, Guangzhou Women and Children's Medical Center, Guangzhou, China

**Keywords:** isolated fetal ventricular septal defect, chromosomal microarray, copy number variants, prenatal diagnosis, postnatal outcome, spontaneous closure

## Abstract

**Objective:**

To evaluate the utility of chromosomal microarray analysis (CMA) in fetuses with isolated ventricular septal defect (VSD) and to explore the favorable factors for predicting spontaneous closure of defects.

**Methods:**

The study included 436 singleton pregnancies seen at a referral prenatal diagnosis center, between January 2016 and May 2020, of which 168 fetuses with isolated VSD were diagnosed in the prenatal setting. VSD was classified as an isolated VSD whether it had ultrasound soft markers or not. All patients underwent testing employing quantitative fluorescent polymerase chain reaction (QF-PCR) and CMA as the first-line genetic detection strategies, mainly in amniotic fluid and umbilical blood samples. Rates of chromosomal abnormalities were compared by subgroups of isolated VSD (muscular or perimembranous). Binary logistic regression analysis was performed to predict the independent determinants of spontaneous closure by 2 years.

**Results:**

Overall, the CMA identified clinically significant copy number variations (CNVs) in 7/168 (4.2%) fetuses and variants of unknown significance (VOUS) in 15/168 (8.9%). Muscular and perimembranous VSDs were found in 53.6 and 46.4%, respectively. Clinically significant relevant subchromosomal aberrations were revealed in seven (9.0%) perimembranous VSDs compared with none in 90 muscular defects (*P* < 0.01). The median initial size of the defect in the muscular VSDs was 2.2(1.8–2.7) mm, as compared to that of 2.8 (2.2–3.2) mm in the perimembranous VSDs group (*p* = 0.000). In muscular vs. perimembranous VSDs, spontaneous closure occurred more frequently and earlier [40.0 vs. 15.4% *in utero* (*p* = 0.000), 61.1 vs. 30.8% at 1-year (*p* = 0.000), and 75.6 vs. 42.3% at 2-year (*P* = 0.000)]. Postnatal surgical closure was warranted in 4/90 (4.4%) of the infants with muscular VSDs, as compared to 29/71 (40.8%) with perimembranous defects (*p* = 0.000). Furthermore, isolated muscular type VSD, smaller defect size, and maternal age of less than 35 years are all positive predictors of spontaneous closure of the defects.

**Conclusion:**

This study highlighted the value of microarray for unbalanced subchromosomal abnormalities in fetuses with isolated VSD, particularly in the perimembranous defects. The detection of an isolated muscular VSD prenatally may be considered a benign or likely benign finding; in contrast, for perimembranous VSD, a prenatal CMA should be offered.

## Introduction

Congenital heart disease (CHD) is one of the commonest congenital malformations worldwide, affecting approximately 3–11 per 1 000 live births and a leading cause of neonatal mortality (1–4). Ventricular septal defect (VSD) represents the most frequent type of CHD, which accounts for 35% of its subtypes in newborns (5). VSD can not only exist in isolation but can also be an intrinsic component of other complicated abnormalities, such as tetralogy of Fallot, univentricular atrioventricular connection, transposition of the great arteries, and aortic coarctation or interruption (6). There are multiple pathological mechanisms leading to VSD, and identification of these mechanisms is necessary for optimal pregnancy management and informed decisions of the parents. Of note, one cause may be the presence of chromosomal abnormalities associated with structural abnormalities, accounting for 26 to 45% of the VSD series (7, 8). However, chromosomal abnormalities may also be present when only ventricular septal defects are detected by prenatal ultrasound screening (9–11). There is also limited evidence on the perinatal evolution of spontaneous closure rates in fetal VSDs within 2 years of age due to the fact that long-term follow-up is nearly non-existent (12, 13).

Chromosomal microarray analysis (CMA), with high resolution and short turnaround time, is known to improve the detection of genomic aberrations and copy number variations (CNVs) compared to conventional karyotyping and has been found to have a pathogenic CNVs detection rate of approximately 6.0% in fetuses with ultrasound structural malformations (14, 15). An increasing number of studies in recent years have employed CMA to identify CNVs in prenatal and postpartum subjects with isolated VSD (12, 16, 17). Although most published reports include large cohorts, investigators have studied isolated VSD as a subset of them, resulting in microscopic samples (18–20). Remarkably, results varied widely, with reported detection rates of pathogenic CNVs ranging from 1.2 to 6.9%, including VSDs with differing definitions of “isolated” (16–21). In addition, subgroup analyses of different types of VSDs, the most typical structural abnormalities detected in the prenatal setting, are rarely reported (22, 23). Therefore, the possible association between isolated VSD and the risk of chromosomal abnormalities, and whether the prenatal invasive procedure should be granted access to pregnant women when an isolated VSD has been detected in the prenatal setting, remains controversial.

This study aims to assess comprehensively the utility and nature of abnormal CMA results in unselected fetuses with isolated VSD and to explore the favorable factors potentially for predicting spontaneous closure of defects throughout a 2-year follow-up period. In particular, the postnatal outcomes and potential diagnostic yields of CMA for different VSD subgroups will also be evaluated.

## Materials and methods

The data comes from a retrospective cohort study that included all singleton fetuses diagnosed with isolated VSD without apparent structural ultrasound abnormalities, regardless of gestational age at onset, and referred to Guangzhou Women and Children's Medical Center between January 2016 and May 2020. The study protocol was approved by the Ethics Committee of Guangzhou Women and Children's Medical Center. All patients had granted their written informed consent for the use of their data in research. This investigation was carried out according to the relevant guidelines and regulations. The inclusion criterion was an isolated VSD diagnosed by echocardiography, and exclusion criteria were multiple pregnancies, maternal age of <18 years old, and diagnosis with ultrasound structural malformation but not ultrasound soft markers at the time of antenatal and postnatal. VSD was identified as an isolated VSD whether it had ultrasound soft markers or not in this study. These soft markers included echogenic foci in the heart or bowel, thickened nuchal folds, absent or hypoplastic nasal bone, single umbilical artery, persistent left superior vena cava, and choroid plexus cysts were not excluded, as these findings would not influence the postnatal cardiac management of the VSD.

Maternal and fetal clinical characteristics and perinatal outcomes were obtained from an electronic ultrasound database and medical records, including maternal age, reproductive history, gestational age at diagnosis, location and size of the VSD, intrauterine or postnatal closure, karyotype and chromosomal microarray results, the outcome of pregnancy, mode of delivery, gestational age at birth, neonatal physical examination, and postnatal treatment if needed. Routine patient follow-up telephone conversations were also used to gather information. Clinical postnatal follow-up assessments were scheduled from birth to 2 years.

The fetal echocardiographic examinations, using grayscale and color Doppler ultrasound, were performed by expert sonographers in evaluating the fetal heart. Within a month of delivery, postnatal echocardiography was repeated on these fetuses to determine whether the ventricular septal defect persisted. All apparently isolated cases of VSD were reviewed to confirm whether VSD was an isolated finding. According to their location, VSDs were observed in the region of the muscular septum, where they were termed to as muscular defects, and within the membranous septum, termed perimembranous defects (24). This method was used both because of the occasional difficulty in making a more accurate assessment of the type of defect prenatally and as it has been employed previously for this purpose (25, 26). Spontaneous closure was defined as the absence of a color flow mapping shunt. The size of the defect was measured by bidimensional imaging unless the boundary could not be precisely spotted; alternatively, the thickness of the colored jet that flows through the septum can be employed, although the latter may lead to an overestimation of the size.

The study population included fetuses diagnosed with isolated VSD who underwent invasive genetic testing with CMA results. Fetal DNA was extracted from amniocytes and umbilical blood by utilizing a Qiagen DNA Blood Midi/Mini Kit (Qiagen GmbH, Hilden, Germany). Invasive samples were analyzed with quantitative fluorescent polymerase chain reaction (QF-PCR) by utilizing a multiplex ligation-dependent probe amplification (MLPA) kit to screen for aneuploidy on chromosomes 13, 18, 21, X, and Y or to rule out maternal cell contamination (Guangzhou Darui Biotechnology Co., Ltd, Guangdong, China). CMA was canceled if the QF-PCR result indicated aneuploidy; in the case of trisomy 13 or 18, or 21, and monosomy X, cytogenetic analysis was carried out in its place. Karyotyping analysis was performed using conventional G-banding techniques (550-band resolution). When the QF-PCR results were normal, CMA was conducted in accordance with the manufacturer's protocol (Affymetrix Inc., Santa Clara, CA, United States), utilizing the Affymetrix CytoScan HD/750K array with a series of single-nucleotide polymorphism array (SNP array) and an array-based comparative genomic hybridization (aCGH) platform at resolutions of 10 and 100 kb, respectively. According to the joint consensus recommendations of the American College of Medical Genetics (ACMG) and Clinical Genome Resource (ClinGen), CNVs were divided into five categories: pathogenic, likely pathogenic, variants of unknown significance (VOUS), likely benign, and benign (27, 28). Genomic coordinates were evaluated following genome build GRCh37/hg19. CMA and karyotype results were reported employing the International System for Human Cytogenomic Nomenclature (ISCN 2020). In addition, DNA taken from maternal and paternal blood samples was typically analyzed in the context of CMA results for heritability assessment or trios analysis.

Pathogenic CNVs, likely pathogenic CNVs, and VOUS are recorded and documented, but likely benign and benign VOUS are not taken into account. All reported CNVs were reviewed by two authors (R.L. and F.L.) to ensure that the classification was adequately updated, according to knowledge described by public databases and gained from prior experience. A number of open databases were applied to classify CNVs, including DECIPHER (http://decipher.sanger.ac.uk/), ClinGen resource (https://www.clinicalgenome.org/), Database of Genomic Variants (DGV, http://dgv.tcag.ca/dgv/app/home), ClinVar (https://www.ncbi.nlm.nih.gov/clinvar/) and University of California Santa Cruz (UCSC, http://genome.ucsc.edu/hg19).

Statistical analysis was performed by using the IBM Statistical Program SPSS 27.0. Continuous variables were displayed as mean ± standard deviations (SD) or median (Q1–Q3). Categorical data were expressed as the number of cases and percentages with 95% confidence intervals (CI), and compared using the Chi-square test or Fisher exact test. Multivariate binary logistic regression analysis was performed for natural VSD spontaneous closure to comprehend the affecting factors from the time of diagnosis to 24 months of age. A *P-*value of < 0.05 was considered statistically significant.

## Results

**Figure 1:**
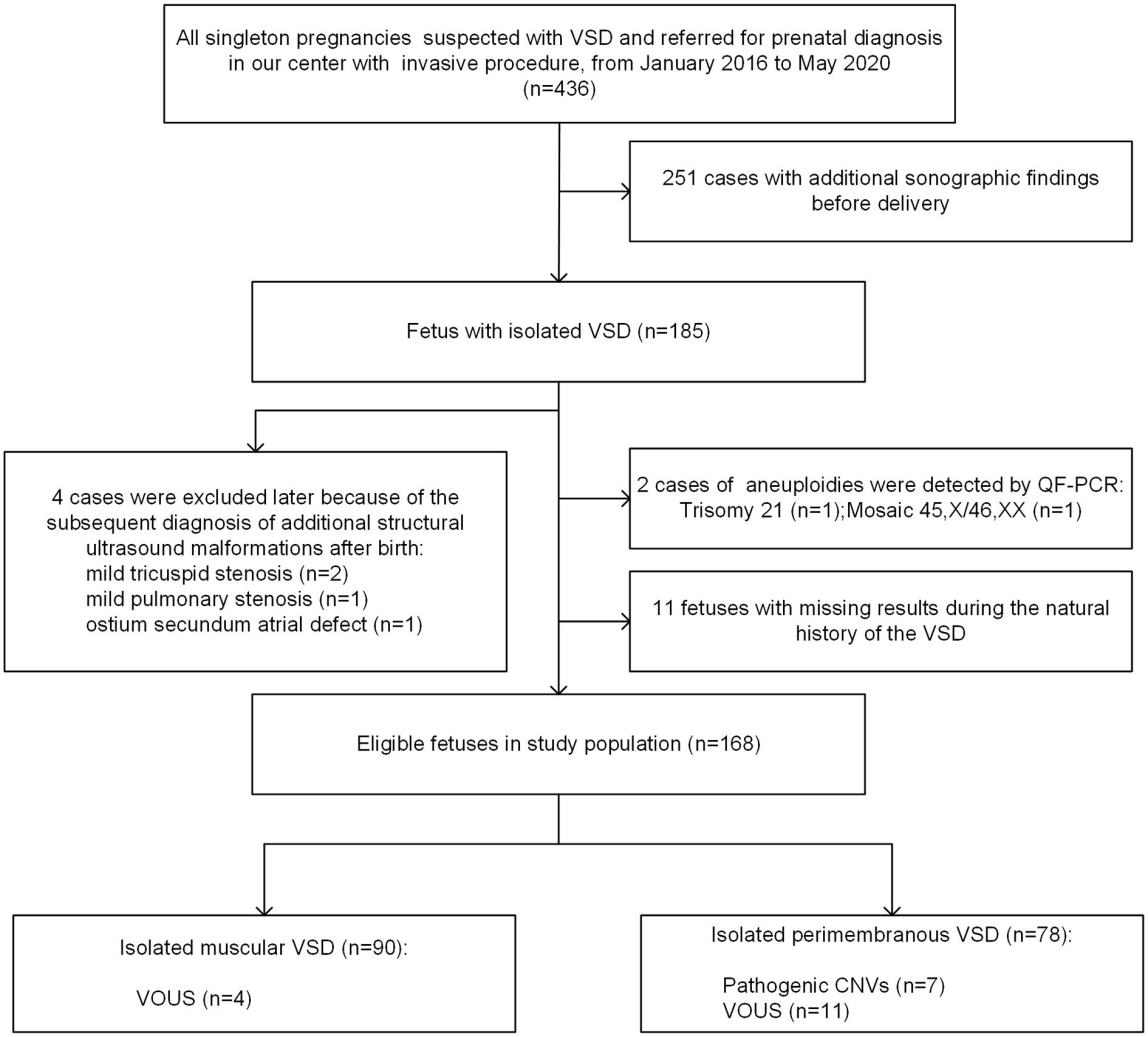
Flowchart of the study population. VSD, ventricular septal defect; QF-PCR, quantitative fluorescent polymerase chain reaction; VOUS, variations of uncertain significance; CNV, copy number variation.

A total of 436 singleton fetuses were diagnosed with VSD and referred for prenatal diagnosis with an invasive procedure in Guangzhou Women and Children's Medical Center from January 2016 to May 2020 (Figure 1). Of these, 185 cases were isolated, whereas 251 pregnancies were associated with additional sonographic findings. From the cohort of 185 isolated VSDs, 4 cases (2.2%) were excluded later due to the subsequent diagnosis of additional structural ultrasound malformations after birth, and 11 fetuses (5.9%) were lost to follow-up during the natural history of the VSD. Furthermore, two chromosomal numerical anomalies were detected by QF-PCR, including one case with trisomy 21 and another with mosaic 45, X/46, XX, both of which were confirmed by karyotyping analysis. Finally, 168 cases of isolated VSDs with complete data during the natural course were analyzed in further detail.

The baseline maternal and neonatal characteristics of the study population are listed in Table 1. The mean maternal age (when reported) was 30.8 ± 4.6 years, and the median gestational age at VSD diagnosis was 25^+1^ (23^+5^-27^+5^) weeks. Among all cases, 130/168 (77.4%) were diagnosed in the late second trimester (20–27^+6^ weeks of gestation), and the remaining 38/168 (22.6%) during the third trimester. Muscular VSD was found in 90 cases (53.6%), whereas 78 fetuses (46.4%) presented with a perimembranous defect. The median initial size of the defect in the muscular VSDs group was 2.2(1.8–2.7) mm, as compared to that of 2.8 (2.2–3.2) mm in the perimembranous VSDs group (*p* = 0.000).

**Table 1:** Maternal and neonatal characteristics of the study population.

	**All subjects** **(*n =* 168)**	**Isolated muscular VSD** **(*n =* 90)**	**Isolated perimembranous VSD** **(*n =* 78)**	***P*-value**
Maternal age (years)	30.8 ± 4.6	30.8 ± 4.2	30.8 ± 5.0	0.952
GA at diagnosis (weeks)	25^+1^ (23^+5^-27^+5^)	25^+0^ (23^+5^-28^+0^)	25^+2^ (24^+0^-27^+3^)	0.766
Initial VSD size (mm)	2.4 (2.0–3.0)	2.2 (1.8–2.7)	2.8 (2.2–3.2)	**0.000**
Primipara	33/168,19.6%	16/90,17.8%	17/78,21.8%	0.513
TOP	7/168,4.2%	0/90,0%	7/78,9.0%	**0.004**
Preterm birth	7/161,4.3%	2/90,2.2%	5/71,7.0%	0.242
Cesarean delivery	57/161,35.4%	27/90,30.0%	30/71,42.3%	0.106
Female sex	88/161,54.7%	48/90,53.3%	40/71,56.3%	0.704
GA at birth (weeks)	39^+2^(38^+5^-40^+0^)	39^+2^(38^+5^-40^+0^)	39^+3^(38^+5^-40^+0^)	0.575
Birthweight (gram)	3,160 (3,000–3,435)	3,180 (3,000–3,300)	3,150 (2,840–3,500)	0.961
Cardiac operation	33/161,20.5%	4/90,4.4%	29/71,40.8%	**0.000**

VSD, ventricular septal defect; GA, gestational age; TOP, termination of pregnancy.

In 113 (67.3%) pregnancies, amniocentesis was performed for genetic diagnosis; and in the remaining 55 (32.7%) cases, percutaneous umbilical blood sampling was conducted. Pathogenic CNVs were found in 7 of 168 fetuses, resulting in an overall detection rate of genetic anomalies by CMA of 4.2% (95% CI 1.1–7.2%). Among the total of 168 fetuses, 15 fetuses (8.9%, 95% CI 4.6–13.3%) were detected with VOUS. By stratified statistical analysis, seven of the 78 perimembranous VSDs (9.0%) compared to none of the 90 muscular defects were associated with chromosomal abnormalities of clinical significance (*P* < 0.01). Additionally, there was a decrease in the detection rate of VOUS in the muscular VSDs group (4.4%, 4/90 vs. 14.1%, 11/78 *p* < 0.05).

Table 2 summarizes the chromosomal characteristics in 7 cases with clinically significant variants as well as clinical and ultrasound data. Among the 7 cases with clinically significant variants, 6 cases were detected with CNVs < 10 Mb, and only one with CNVs > 10 Mb. The phenotype involved in these CNVs included 22q11.2 deletion syndrome (*n* = 3), 4p16.3 deletion syndrome (*n* = 1), 11q24.2 deletion (*n* = 1), 1q21.1 duplication syndrome (*n* = 1), and 16p13.11 duplication (*n* = 1). Interestingly, all of these found clinically significant variants were perimembranous defects. A total of 7 pregnancies chose to terminate the pregnancy after being informed of the abnormal chromosomal results. The information on chromosomal data of VOUS as well as clinically relevant characteristics is shown in Supplementary Table S1. Only about a quarter of fetuses with ventricular septal defects that do not involve soft ultrasound markers subsequently develop spontaneous closure.

**Table 2:** Clinically relevant characteristics of isolated VSD fetuses and clinically significant CMA findings.

**Case number**	**GA at diagnosis of VSD** **(weeks)**	**Invasive procedure**	**Ultrasound soft markers**	**Size** **(mm)**	**Type of VSD**	**Microarray results**	**Type of CNV**	**Length** **(Mb)**	**Interpretation**	**Outcome**	**Parental study**
1	25 + 6	AC	Hypoplastic nasal bone	2	Perimembranous	arr[hg19]4p16.3p15.33(68345_14195870) ×1	Deletion	14.13	Pathogenic	TOP	de novo
2	23 + 3	AC	Echogenic intracardiac focus	1.8	Perimembranous	arr[hg19]22q11.21q11.23(21465661_23810042) ×1	Deletion	2.34	Pathogenic	TOP	de novo
3	26 + 2	AC	PLSVC	2.7	Perimembranous	arr[hg19]11q24.2q25(126039017_134938470) ×1	Deletion	8.90	Pathogenic	TOP	NA
4	28 + 4	PUBS	NO	3.5	Perimembranous	arr[hg19] 22q11.21(18648866_21465662) ×1	Deletion	2.82	Pathogenic	TOP	*de novo*
5	27 + 1	AC	NO	4.2	Perimembranous	arr[hg19]1q21.1q21.2(146488131_147819294) ×3	Duplication	1.33	Pathogenic	TOP	Paternally inherited
6	23 + 0	PUBS	Choroid plexus cysts	3.8	Perimembranous	arr[hg19] 16p13.11(15140210_16326223) ×3	Duplication	1.19	Pathogenic	TOP	*de novo*
7	24 + 4	PUBS	NO	4.5	Perimembranous	arr[hg19] 22q11.21(18648855_21800471) ×1	Deletion	3.15	Pathogenic	TOP	*de novo*

VSD, ventricular septal defect; CMA, chromosomal microarray analysis; CNV, copy number variation; GA, gestational age; AC, amniocentesis; TOP, termination of pregnancy; PLSVC, persistent left superior vena cava; PUBS, percutaneous umbilical blood sampling.

Forty-eight (28.6%) of 168 defects closed spontaneously *in utero*, 79 (47.0%) defects closed spontaneously within 1-year and in 101 (60.1%) at 2-year, whereas in 67 (39.9%) cases, the VSD remained patent. Spontaneous closure was higher frequency and earlier in muscular vs. perimembranous VSDs [40.0 vs. 15.4% *in utero* (*p* = 0.000), 61.1 vs. 30.8% at 1-year (*p* = 0.000), 75.6 vs. 42.3% at 2-year (*P* = 0.000)]. The rate of spontaneous closure by the VSD site at different stages is given in Table 3. In a nutshell, spontaneous intrauterine closure occurred more frequently in muscular VSDs, while spontaneous closure in the postnatal stage was observed mainly in infants with perimembranous defects. The postnatal cardiac operation was warranted in 4/90 (4.4%) of the infants with a muscular VSDs, as compared to 29/71 (40.8%) with a perimembranous VSDs (*p* = 0.000).

**Table 3:** Rate of spontaneous closure of isolated VSD at different stages according to different types of defects.

**Type of VSD**	**Spontaneous closure**			**No closure**	**Total**
	**Intrauterine**	**12 months**	**24 months**		
Muscular	36 (40.0)	55 (61.1)	68 (75.6)	22 (24.4)	90 (53.6)
Perimembranous	12 (15.4)	24 (30.8)	33 (42.3)	45 (57.7)	78 (46.4)
Total	48 (28.6)	79 (47.0)	101 (60.1)	67 (39.9)	168 (100)

VSD, ventricular septal defect; data given as *n* (%).

**Figure 2:**
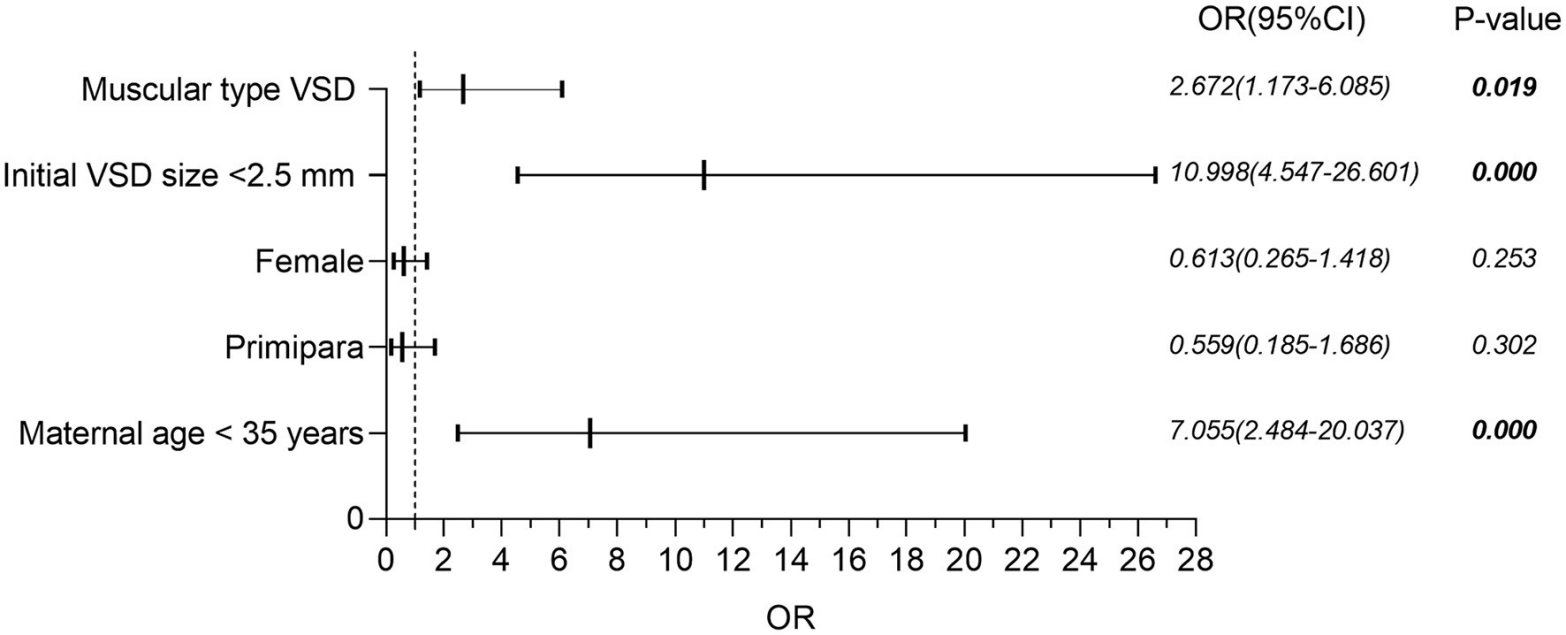
Binary logistic regression analysis of factors potentially affecting the risk of spontaneous closure in isolated VSD. VSD, ventricular septal defect; OR, odds ratio; CI, confidence interval.

Binary logistic regression analysis was performed to determine the independent contributions of the location of VSD (muscular or perimembranous), the initial size of VSD (<2.5 or ≥2.5 mm), and maternal age (<35 or ≥35 years) in the prediction of spontaneous closure before the age of 2 years. Figure 2 shows that isolated muscular VSD, the initial size of VSD less than 2.5 mm, and maternal age less than 35 years are all favorable prognostic indicators to predict the spontaneous closure of VSD from the time of diagnosis until 24 months of age. Neither the gender of the fetus nor whether those pregnant women were primiparous, was a statistically significant factor.

## Discussion

In this study, we describe the prevalence and distribution of genetic variants for different types of VSDs and explore potential favorable factors for the prediction of spontaneous closure of defects. Our study confirms that the value of chromosomal microarray for unbalanced genomic variants in fetuses with isolated VSDs, particularly in the perimembranous VSDs group. Prenatally isolated muscular VSD is not associated with clinically significant chromosomal abnormalities, and with a favorable clinical outcome, as well as a low risk of needing a cardiac operation after birth. In comparison, isolated perimembranous VSD increases the risk of chromosomal aberrations and is more likely to require treatment after delivery. Isolated muscular type VSD, smaller defect size, and maternal age of less than 35 years are all positive predictors of spontaneous closure of the defect. These objective data proposed may be helpful in counseling patients about isolated VSD detected prenatally.

According to our data, 90 (53.6%) muscular VSDs and 78 (46.4%) perimembranous defects were diagnosed, comparable to the two previous extensive reports (29, 30). There is much disagreement in the literature regarding the association between isolated VSDs and chromosomal aberrations. Some reports suggest the association is low (16, 17, 20), while other studies point to an increased risk of chromosomal abnormalities (19, 21). However, these studies either had small sample sizes or varied inclusion criteria, with no uniform definition of “isolated” VSDs, while some excluded cases with high-risk assessment in the first trimester, which may have changed the composition of second trimester fetuses presented for the survey. The choice of different resolution platforms and whether to utilize the latest version of the joint consensus recommendations of the ACMG and ClinGen are equally noteworthy. The total diagnostic yield of submicroscopic genetic abnormalities defined as pathogenic and likely pathogenic CNVs by CMA in this study was 4.2%, slightly lower than in previous work at our center (5.5%) (18). Our investigation also found that the incidence of genetic variation in fetuses with isolated perimembranous VSD was significantly higher than that in fetuses with muscular defects (9.0 vs. 0%, *P* < 0.01). Gómez et al. (9) stratified their results according to the type of VSD, finding that no chromosomal abnormalities of clinical significance were discovered in 216 fetuses with isolated muscular VSD. In the same vein, another study focused only on isolated muscular defects diagnosed prenatally revealed that isolated muscular VSD was neither associated with a significant increase in the prevalence of chromosomal abnormalities nor with the incidence of pathogenic or likely pathogenic CNVs (23). Therefore, it seems that invasive prenatal testing should be recommended in a series of isolated perimembranous VSD.

In addition, the overall proportion of VOUS was 8.9% (15/168), which was higher than the frequency reported in previous studies (18, 19). These discordant findings could be explained, at least in part, by differences in sample size, database platform, and trio analysis. Of note, VOUS, especially in proband-only samples, will complicate prenatal counseling and parental decision-making. In such cases, the risk of generating parental anxiety must be weighed against the need for expensive (closer to $1,000) tests. However, with the explosion in the use of CMA in genetic disorders, the interpretation of more and more CNVs data has evolved over time, which is expected to reduce the number of VOUS in the future significantly.

VSD without any malformation could be the only sign of some microdeletions and micro-duplication syndromes. Of all our genetic aberration results, 22q11.2 microdeletion, in particular, was found to be remarkably more common in fetuses with isolated VSD. A previously published report by Park et al. (31) found that isolated VSDs could be found in up to 20.5% of patients with a chromosome 22q11.2 deletion. Besides 22q11.2 deletion syndrome [Online Mendelian Inheritance in Man (OMIM) #611867], we also identified some classical CNVs, including Wolf-Hirschhorn syndrome (OMIM #194190), Jacobsen syndrome (OMIM #147791), and 1q21.1 duplication syndrome (OMIM #612475). These syndromes manifest with a range of physical and mental disabilities and congenital cardiac malformations, including VSD. Moreover, 16p13.11 duplication size of 1.19 Mb (case 6), characterized by variable expression and incomplete penetrance, with the most common clinical features were speech delay (88%) and intellectual deficiency (86%), and cardiac malformations found in 23% of patients (32).

In terms of clinical outcomes, the rate and timing of spontaneous closure was 28.6% *in utero*, 40.0% at 1-year, and 60.1% of all defects at 2-year follow-up, a finding similar to previous observations (10, 33). In our series, the intrauterine natural closure rate of muscular VSDs was 40.0%, with that of perimembranous defects at 15.4%. Although previous studies reported that 27.3–54.1% of all prenatally detected perimembranous VSDs closed spontaneously *in utero*, these studies consisted of smaller cohorts (9, 12, 22, 30). As reported in the literature, this high incidence of spontaneous closure of muscular VSD can be seen as a normal process of delayed, underlying physiological development, rather than an abnormality (34). The incidence of spontaneous VSD closure varies widely, depending on the age and gender of the subject, the size and type of the defect, the population studied, as well as the length of follow-up (35). It is hypothesized that this relationship between VSD type and spontaneous closure is related to the different closure mechanisms of each VSD. The mechanism of spontaneous closure of muscular VSD is thought to be the localization of muscular tissue or fibrous tissue formation on the right ventricular side; however, in rare cases, aneurysm formation of fibrous tissue is involved (36). On the other hand, perimembranous defects can be closed by tricuspid valve aneurysm formation or prolapse of the right aortic cusp (35). Additionally, a large study published in 2020 by Cox et al. (29) included 177 muscular and 162 perimembranous VSDs and found that 4% of muscular and 47% of perimembranous defects required surgical closure, which is consistent with what we found. These further strengthen the conclusion that perimembranous VSD has an impact on morbidity compared with muscular defects.

It is logical to consider that the size of the VSD is inversely proportional to the likelihood of natural closure. Although the factors predicting spontaneous closure of the defect appear to be a further independent clinical issue, it is nevertheless part of the natural course of fetal diagnosed isolated VSD. The favorable predictors of spontaneous closure of the defect, in other words, the risk factors for VSD persistence, are also demonstrated. Most of the parents endure severe anxiety and guilt for their fetal heart anomaly. However, isolated VSD is successfully curable in most cases. Therefore, it is crucial to calculate indicators that can predict the natural closure of VSD, and these should be presented without exaggeration during consultation. Regarding the index of the possibility of spontaneous closure of VSD, we could confirm previous observations (11, 30, 37), indicating that isolated muscular VSD, the initial size of VSD less than 2.5 mm, and maternal age less than 35 years are essential predictors of natural closure. As for the association between fetal gender and whether it is primiparous and the spontaneous closure of the VSD, however, more studies will be needed to find out more details. In short, these findings could facilitate more effective parental counseling by providing parents with a clearer picture of the expected outcomes.

We acknowledge that our study has several limitations. Firstly, this study is retrospective, which has inherent limitations, and prospective studies with a larger cohort are needed. Secondly, data from a single-center and a lack of different ethnic backgrounds, however, this study has less heterogeneity. Thirdly, our detection could overlook balanced chromosomal rearrangements. Finally, although no manifestations of neurodevelopmental abnormalities were reported, we followed them up at only 24 months of age. A longer follow-up period may be required in childhood to detect subtle neurodevelopmental abnormalities.

## Conclusion

In conclusion, our study emphasized the value of microarray for unbalanced submicroscopic chromosomal abnormalities in fetuses with isolated VSD, particularly in the perimembranous VSDs group. The diagnosis of prenatal isolated muscular VSD can be considered a benign or likely benign finding, with no cases of clinically significant subchromosomal anomalies, associated with favorable postpartum outcomes and rarely requiring surgery after birth. However, perimembranous defects increase the risk of chromosomal aberrations, and prenatal invasive CMA testing should be recommended all the more. The data also suggested that factors such as the type of VSD, initial VSD size, and maternal age can influence the rate of spontaneous closure. The information supplied here, we hope, will contribute to more effective parental counseling and professional clinical management of isolated VSD detected prenatally.

## Data availability statement

The original contributions presented in the study are included in the article/Supplementary material, further inquiries can be directed to the corresponding author.

## Ethics statement

The studies involving human participants were reviewed and approved by Ethics Committee of the Guangzhou Women and Children's Medical Center. The patients/participants provided their written informed consent to participate in this study. Written informed consent was obtained from the individual(s) for the publication of any potentially identifiable images or data included in this article.

## Author contributions

KC, HZ, FF, RL, and FL performed the research study and analyzed the data. DL, XY, and TL provided clinical data. KC and HZ prepared the figures and the tables. YW and RH collected the data. KC and CL wrote the manuscript. All authors contributed to the article and approved the final manuscript.

## Funding

This work was funded by the sub-project of the National Key Research and Developmental Program (Grant No. 2021YFC2701002), the National Natural Science Foundation of China (Grant Nos. 81801461, 81873836, 81771594, 81671474, and 81501267), the Natural Science Foundation of Guangdong Province (Grant Nos. 2019A1515012034 and 2017A030313460), and the Project of Guangzhou Science and Technology (Grant No. 202102020191).

## Conflict of interest

The authors declare that the research was conducted in the absence of any commercial or financial relationships that could be construed as a potential conflict of interest.

## Publisher's note

All claims expressed in this article are solely those of the authors and do not necessarily represent those of their affiliated organizations, or those of the publisher, the editors and the reviewers. Any product that may be evaluated in this article, or claim that may be made by its manufacturer, is not guaranteed or endorsed by the publisher.
