# Supplementary material for: Should prenatal chromosomal microarray analysis be offered for isolated ventricular septal defect? A single-center retrospective study from China
Source: Front Cardiovasc Med. 2022 Sep 7;9:988438. doi: 10.3389/fcvm.2022.988438 (PMC9489942; doi:10.3389/fcvm.2022.988438)
Supplement: Supplementary file 1 [file Table_1.docx]

Supplementary Table S1 Clinically relevant characteristics of isolated VSD fetuses and chromosomal data of VOUS

| Case number | GA at diagnosis of VSD  (weeks) | Invasive procedure | | Ultrasound soft markers | Size  (mm) | Type of VSD | Microarray results | Type of CNV | Length | Fetal sex | Outcome | Parental study |
| --- | --- | --- | --- | --- | --- | --- | --- | --- | --- | --- | --- | --- |
| 8 | 23+2 | AC | NO | | 2.3 | muscular | arr[hg19]3q29(193228047_194767726)×3 | duplication | 1.54Mb | Male | Spontaneous closure | Maternally inherited |
| 9 | 24+1 | AC | NO | | 2.8 | muscular | arr[hg19]4q35.2(187922671_188948946)×3 | duplication | 1.03Mb | Male | Spontaneous closure | NA |
| 10 | 24+3 | PUBS | NO | | 2.9 | muscular | arr[hg19]14q31.3(86083278_86617981)×1  arr[hg19]Xq11.2(63836582_64335987)×2 | deletion  duplication | 535Kb  499Kb | Female | No closure | NA |
| 11 | 30+6 | PUBS | NO | | 2.5 | muscular | arr[hg19]4q13.3q21.1(75606645_76725031)×3 | duplication | 1.12Mb | Male | Spontaneous closure | de novo |
| 12 | 25+4 | AC | NO | | 1.9 | perimembranous | arr[hg19]4q35.2(188842353_189939305)×3 | duplication | 1.10Mb | Male | Spontaneous closure | NA |
| 13 | 24+6 | AC | NO | | 2.9 | perimembranous | arr[hg19]3p12.3p12.2(79308172_80416280)×3 | duplication | 1.11Mb | Female | Cardiac operation | NA |
| 14 | 24+3 | AC | PLSVC | | 2.5 | perimembranous | arr[hg19]21q11.2(15006457_15713941)×3 | duplication | 707Kb | Female | No closure | Paternally  inherited |
| 15 | 24+2 | AC | NO | | 2 | perimembranous | arr[hg19]16p13.11p12.3(16388172_16881097)×3 | duplication | 493Kb | Female | No closure | NA |
| 16 | 26+1 | AC | NO | | 2 | perimembranous | arr[hg19]11q14.1q14.2(79532293_86583442)×2 | LOH | 7.05Mb | Male | Cardiac operation | de novo |
| 17 | 25+0 | AC | Echogenic intracardiac focus | | 2.7 | perimembranous | arr[hg19]21q21.1q21.2(23916500_26312625)×3 | duplication | 2.40Mb | Female | Cardiac operation | NA |
| 18 | 23+3 | AC | Choroid plexus cysts | | 4.6 | perimembranous | arr[hg19]4q28.3q31.1(139282081_139541253)×3 | duplication | 259Kb | Female | Cardiac operation | de novo |
| 19 | 25+5 | PUBS | Hypoplastic nasal bone | | 3.2 | perimembranous | arr[hg19]2q13(110504318_111370025)×1 | deletion | 866Kb | Female | Cardiac operation | de novo |
| 20 | 24+1 | PUBS | NO | | 3.3 | perimembranous | arr[hg19]2q13(110496601_111365996)×3 | duplication | 869Kb | Female | No closure | NA |
| 21 | 26+5 | AC | NO | | 2.4 | perimembranous | arr[hg19]22q11.21(18505505_19004731)×3 | duplication | 499Kb | Female | Cardiac operation | NA |
| 22 | 31+4 | PUBS | NO | | 3.2 | perimembranous | arr[hg19]11q24.1q24.2(123850536_124306215)×3 | duplication | 456Kb | Male | Cardiac operation | de novo |

VSD, ventricular septal defect; VOUS, variant of unknown significance; GA, gestational age; CNV, copy number variation; AC, amniocentesis; NA, not available; PUBS, percutaneous umbilical blood sampling; PLSVC, persistent left superior vena cava; LOH, loss of heterozygosity.
